# Managing the Increasing Burden of Atrial Fibrillation through Integrated Care in Primary Care: A Cost-Effectiveness Analysis

**DOI:** 10.5334/ijic.5661

**Published:** 2023-05-03

**Authors:** Carline J. van den Dries, Miriam P. van der Meulen, Geert W. J. Frederix, Arno W. Hoes, Karel G. M. Moons, Geert-Jan Geersing

**Affiliations:** 1 Julius Center for Health Sciences and Primary Care, University Medical Center Utrecht and Utrecht University, Str. 6.131, PO Box 85500, 3508 GA Utrecht, the Netherlands

**Keywords:** integrated care, atrial fibrillation, primary care, cost-effectiveness, multimorbidity

## Abstract

**Introduction:**

Integrated care for patients with atrial fibrillation (AF) in primary care reduced mortality compared to usual care. We assessed the cost-effectiveness of this approach.

**Methods:**

Dutch primary care practices were randomised to provide integrated care for AF patients or usual care. A cost-effectiveness analysis was performed from a societal perspective with a 2-year time horizon to estimate incremental costs and Quality Adjusted Life Years (QALYs). A sensitivity analysis was performed, imputing missing questionnaires for a large group of usual care patients.

**Results:**

522 patients from 15 intervention practices were compared to 425 patients from 11 usual care practices. No effect on QALYs was seen, while mean costs indicated a cost reduction between €865 (95% percentile interval (PI) –€5730 to €3641) and €1343 (95% PI –€6534 to €3109) per patient per 2 years. The cost-effectiveness probability ranged between 36% and 54%. In the sensitivity analysis, this increased to 95%-99%.

**Discussion:**

Results should be interpreted with caution due to missing information for a large proportion of usual care patients.

**Conclusion:**

The higher costs from extra primary care consultations were likely outweighed by cost reductions for other resources, yet this study doesn’t give sufficient clarity on the cost-effectiveness of integrated AF care.

## Background

Atrial fibrillation (AF) is the most common heart rhythm disorder with a prevalence that increases with age, up to 17.8% in patients aged 85 years and above [1]. Thus, with the ageing population, the population-wide prevalence of AF will increase even further. Indeed, the number of AF patients is expected to more than double between the years 2010 and 2060 [2]. AF is a chronic condition associated with multiple comorbidities [3]. Thus, multiple caregivers are often involved – such as cardiologists, anticoagulation specialists, geriatricians, general practitioners, practice nurses and home care providers– which induces a risk of fragmented care. Furthermore, AF is associated with high healthcare expenditures. Hospital admissions occur very frequently and are an important cost-driver, accounting for 50–70% of all AF-related costs [4,5]. Direct annual costs per AF patient vary from €450 to €3000 in Western Europe [4]. In the Netherlands, direct annual costs for AF patients accounted for €583 million in 2009, reflecting 1.3% of the Netherlands healthcare expenditure [6]. With the increasing prevalence of AF, total costs and burden on health care resources will likely increase as well, emphasising the urgent need to investigate other, more (cost-)effective ways to organise care for AF patients.

As is described in the European Society of Cardiology guidelines on the management of AF, one potential solution could be ‘integrated care’, i.e. coordinated and optimized patient-individualized care through a multidisciplinary team [7]. A meta-analysis of studies investigating such integrated care coordinated by hospitals showed a reduction in all-cause mortality and cardiovascular hospitalisation [8]. Furthermore, providing nurse-led integrated care at specialised and experienced AF clinics likely also saves costs [9]. Nevertheless, these studies were all organised from a hospital care setting, whereas many elderly AF patients are no longer managed in outpatient cardiology clinics, but in the primary care setting. Therefore, primary care forms an interesting base to orchestrate integrated AF care from, specifically for the elderly AF population, with the potential also to be more cost-effective.

To quantify the effects of integrated AF care in primary care, we performed the large ALL-IN cluster randomised trial in the Dutch primary healthcare setting. Patients in the index group received a proactive, patient-centred, multidisciplinary integrated care intervention, consisting of i) quarterly check-ups for AF with a focus on treatment of comorbidities, ii) anticoagulation management in primary care, and iii) close collaboration with secondary care [10]. In patients who received this index intervention, we observed a 45% reduction in all-cause mortality when compared to patients receiving usual care [11]. Analysis of the cost-effectiveness of this intervention was a secondary objective of the ALL-IN trial. This paper describes the potential cost-effectiveness of organising integrated care for AF patients in primary care. If proven cost-effective, integrated care with its basis in primary care could be instrumental in tackling the urgent public health challenge of AF.

## Methods

### Study design of the ALL-IN trial

The study design of the ALL-IN trial was described in detail previously [10]. In addition, a detailed comparison of the intervention with usual care is provided in the Additional file, Table A1. In short, we performed a cluster-randomised, pragmatic, non-inferiority trial in primary care practices in the Netherlands, starting in 2016 with a follow-up period of 2 years. After randomisation of primary care practices, patients with documented AF aged 65 years or older were included. The main exclusion criteria were valvular AF or the presence of an internal cardioverter defibrillator or cardiac resynchronisation therapy device [10]. In practices randomised to the index intervention, patients who provided informed consent for participating in the intervention received integrated care and also a questionnaire on quality of life and resource use at baseline, after 12 months and after 24 months of follow-up. A modified informed consent procedure was carried out, in which a waiver for informed consent to collect data on baseline characteristics and clinical outcomes from the primary care electronic medical records (EMRs) was provided by the Medical Ethics Committee of the Isala Clinics Zwolle [10,11]. Such a waiver of informed consent for anonymised data collection was necessary to ensure the scientific validity of the cluster randomised trial, for three reasons: (i) to assess otherwise undetectable possible selection bias caused by providing individual informed consent for participation *after* cluster randomization, (ii) to enhance the generalizability of the results, especially to frail elderly AF patients, and (iii) informing the eligible patients in the usual care practices about the aims of the study would imply patient education about AF and its risks, thus inducing a risk of contamination. Patients in control practices were asked for informed consent to fill out the questionnaires on quality of life and resource use. The ALL-IN trial is registered at the Netherlands Trial Register (NL5407).

### The integrated care intervention

The multidisciplinary index intervention was based on the “Components of High-Quality Chronic Illness Care” developed by Wagner *et al* [12] and consisted of three main aspects: (i) quarterly check-ups by a primary care practice nurse supervised by the GP, for AF and its related comorbidities, including patient education and detection of early signs and symptoms of heart failure, (ii) case management of anticoagulation treatment, with International Normalized Ratio (INR) measurements performed in the primary care practice (or at home if necessary) in patients treated with a vitamin K antagonist (VKA) and special attention for drug compliance and monitoring of kidney function in patients with a non-vitamin K oral anticoagulant (NOAC), and (iii) easy-access consultation and close collaboration with anticoagulation clinics – serving as a “back-office” creating VKA dosage calendars based on the INR measurements received from the primary care practice – and cardiologists and/or cardiac nurses in secondary care. When patients needed to be referred to secondary care or needed additional check-ups by a cardiologist, this was complementary to the check-ups in primary care, ensuring continuity of care. Practice nurses were trained in anticoagulation treatment and monitoring, and educated in the signs, symptoms and treatment of AF and its comorbidities. The training and the protocol for the quarterly check-ups were based on the Dutch College of General Practitioners’ guidelines on AF [13]. Throughout the 2-year follow-up period, 3 multidisciplinary meetings were organised to discuss complex patients and practical issues and provide additional education based on questions from the intervention practices.

In practices randomised to the control group, patients received usual care. Usual care could vary per patient, but mostly consisted of care provided by cardiologists (generally once a year, except for patients who had already been referred back to primary care by their cardiologist), anticoagulation clinics, and ad-hoc consultation of the GP. Some patients were also seen by a practice nurse for treatment of diabetes mellitus type 2, cardiovascular risk management, or chronic obstructive pulmonary disease (COPD), yet without special attention for AF.

### Cost-utility analyses

The outcomes of the cost-utility analysis are the incremental costs and incremental Quality Adjusted Life Years (QALYs). The cost-utility analysis was performed from a societal perspective, so including available costs from different providers and settings, also outside the hospital. The time horizon used was equal to the study period, i.e. 24 months. Given the short follow-up period, discounting of costs and effects was considered redundant. The CHEERS checklist was used to include all applicable elements of a single study-based economic evaluation [14].

### Resource use

Empirical study data were collected for six different cost categories: 1) costs made in primary care practices, 2) costs from cardiology outpatient clinic visits, 3) costs from hospital or nursing home admissions and electrocardioversion (ECV), 4) costs from anticoagulant management, 5) other direct costs, and 6) indirect costs (informal care). As all patients were aged 65 years or older, we did not include productivity losses. The methods to obtain data on resource use are described below.

#### Primary care practices

The number of procedures in primary care were derived from the EMRs of the practices in which the ICT system allowed for such data extraction. Procedures consisted of consultations with GPs and practice nurses and diagnostic/therapeutic procedures (for example surgical procedures by the GP and electrocardiography).

#### Outpatient cardiology visits

For cardiology outpatient clinic visits, patients were asked through the resource use questionnaires administered at 12 and 24 months of follow-up how often, on average, they visited their cardiologist per year. If missing, information on follow-up frequency from the available cardiologist letters in the EMR was used.

#### Admissions and ECV

Information on hospital and nursing home admissions and ECV therapy was collected from specialists’ letters available in the EMRs of the primary care practices. An admission was defined as an admission with at least one overnight stay. For nursing home admissions, only temporary admissions were included in this category, as patients were censored when permanently admitted to a nursing home. Permanent nursing home admissions were taken into account in an additional analysis (see section on statistical analyses).

#### Anticoagulation management

For patients using a vitamin K antagonist in the intervention group, data on the number of INR measurements in 2017 were derived from the three anticoagulation clinics located in the areas of the participating primary care practices. Patients included in the usual care group could not exactly be identified by the anticoagulation clinics [10]. Therefore, the number of INR measurements in 2017 from a representative proxy was taken, including all patients with AF aged 65 years and over, without an artificial heart valve, registered with the affiliated control practices of their region. For simplicity, the anticoagulant used at baseline was assumed to remain unchanged throughout the follow-up period. For vitamin K antagonists, we assumed an average number of 2 tablets acenocoumarol per day.

#### Other direct costs

Through the questionnaires at 12 and 24 months of follow-up, self-reported data on use of the following resources were collected: visits to non-cardiology specialists’ outpatient clinics; emergency department visits, ambulance rides; day admissions (e.g. for short surgical procedures); paramedical care; and home care (by professional caregivers). The answers from the three month recall periods were extrapolated to the follow-up period of 24 months. Data on which patients were living in an assisted living facility were provided by the practices at the end of follow-up.

#### Indirect costs

Resource use of self-reported informal care, was also derived from the questionnaires at 12 and 24 months, and trimmed at 2 hours a day.

### Unit costs

The number of procedures were multiplied by the costs, which were specified in the Dutch Manual for costing research in health care [15]. Missing procedures were obtained from the EMRs of the practices. Costs of anticoagulant drugs were derived from the website www.medicijnkosten.nl. For NOAC treatment, the average price of the four available NOACs was taken and standard doses were assumed. For VKA monitoring, €17,00 per INR measurement was counted [16]. Costs of 2017 were used or updated to 2017 using the consumer price index (CPI).

### Quality Adjusted Life Years (QALYs)

QALYs were calculated using an area under the curve approach. Utility scores were derived from the generic health related quality of life EuroQol 5D-5L questionnaires (EQ5D) filled out by the patients at baseline, after 12 months and after 24 months of follow-up.

### Nursing home admissions

As we could not collect additional follow-up data from nursing homes when patients permanently moved to a nursing home, and because the primary care practice is no longer involved in providing care for these patients, we had to censor patients after a permanent move to a nursing home. Nevertheless, nursing home admission is an important cost-driver and we did collect data on the exact timing of nursing home-admission. Therefore, we performed additional analyses in which we assumed a scenario with the largest impact on costs and QALYs: we assumed these patients survived in the nursing home up to the end of the 2-year follow-up, at a quality of life comparable to a comatose state (utility of 0.1). In this way, the analyses with and without taking permanent nursing home admission into account contribute to the range that likely covers the ‘true’ incremental costs and effects.

### Statistical analyses

Multiple imputation was performed for missing data from the questionnaires, i.e. other direct costs, indirect costs and EQ5D time points (i.e. at baseline, after 12 months and after 24 months). The variables age, sex and Frailty Index (FI, a validated frailty indicator, [17]), death, total GP costs, total admissions and ECV costs and available EQ5D values were used as predictors. Missing data for the number of primary care consultations were not imputed, as the reason for being missing was considered missing completely at random, i.e. depending on the primary care ICT system. Healthcare procedures of patients who died during the study were collected until their date of death from the EMRs of the practices, while healthcare consumption questionnaires were either used or imputed until death. In the analyses, standard deviations were given and/or bootstrapped p-values and bootstrapped percentile intervals (containing 95% of repeats).

In all analyses, costs were adjusted for baseline differences in age, sex, FI and clustering (at the practice level) using multiple regression models. QALY contribution was additionally adjusted for baseline EQ5D-5L utility score. We performed bootstrapping with 100 iterations on each of the 40 imputation sets in order to assess the uncertainty around the incremental costs and effects. The incremental costs and effects of all bootstraps were plotted in cost-effectiveness planes. All analyses were performed using R Statistical Software (Foundation for Statistical Computing, Vienna, Austria).

### Sensitivity analyses

**Figure 1:**
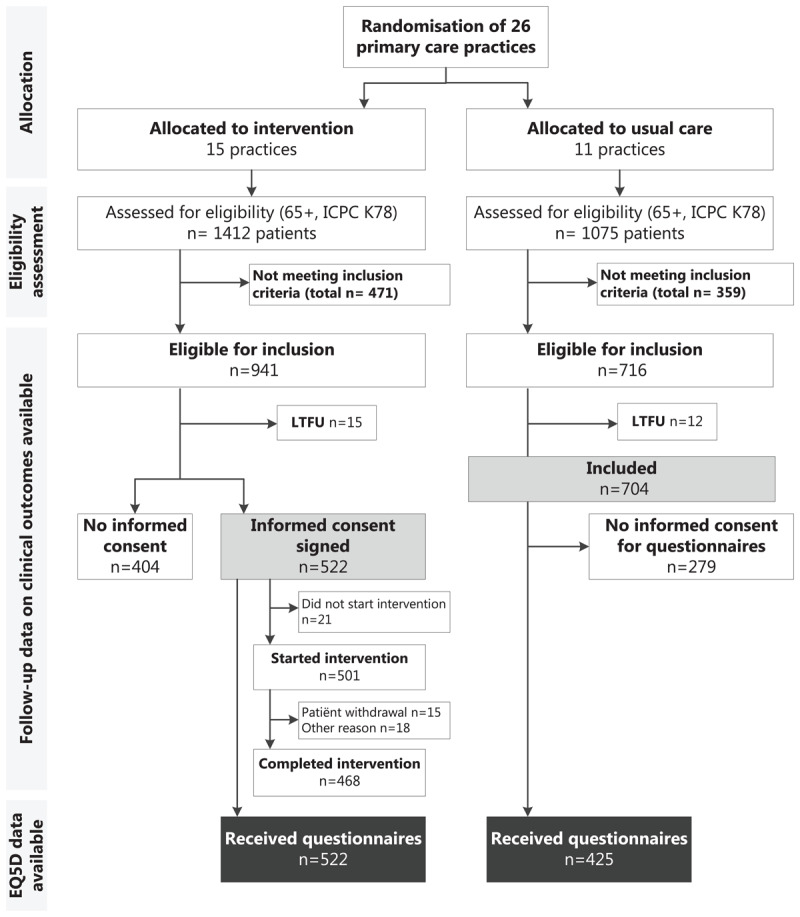
**Flowchart of the ALL-IN trial.** LTFU = Lost to follow-up.

As can be seen in Figure 1, 279 out of 704 eligible usual care patients did not provide informed consent for filling out the questionnaires. For these 279 patients, data for EQ5D and self-reported resource use (denoted with * in Table 2) were missing. However, we did have data on all the other costs of these patients, so we performed a sensitivity analysis comparing all 704 usual care patients to the 522 intervention patients, in which we imputed the missing data from the questionnaires. Here, multiple imputation was not possible for each type of self-reported resource use but was performed on the total costs of other direct and indirect costs, and the missing EQ5D values at the different time points.

In a second sensitivity analysis, a healthcare perspective instead of the societal perspective was applied, disregarding informal care and using unit costs for primary care consultations as specified by the Dutch Health Authority, in which the unit cost per consultation is lower and the residual costs are reimbursed separately through a fixed price per registered patient [18]. The differences in unit costs between these perspectives are shown in the Additional file, Table A2. Finally, a sensitivity analysis was performed with the unadjusted values.

## Results

### Descriptive statistics

15 practices were allocated to the intervention and 11 to the control group (see Figure 1). In the intervention practices, 522 (55.0%) of the eligible patients provided informed consent for participation in the intervention (and for the questionnaires). These 522 patients were included in our analyses and compared to the 425 usual care patients who were willing to fill out detailed questionnaires on healthcare related costs and quality of life.

Baseline characteristics of the 522 intervention patients and the 425 usual care patients are shown in Table 1. An additional column with the 704 usual care patients is shown in the Additional file, Table A3. Most baseline characteristics of the 425 usual care patients willing to fill out questionnaires were more comparable to the 522 intervention patients, than the baseline characteristics of all 704 usual care patients were.

**Table 1:** **Baseline characteristics of included patients.** Numbers are counts (%) unless stated otherwise. The frailty index consists of the presence or absence of 36 health deficit items (scale 0–1, higher value indicating more frailty). EQ5D-5L, EuroQol 5D questionnaire; IQR, interquartile range; NOAC, non-vitamin K antagonist oral anticoagulant; RAAS, renin–angiotensin–aldosterone system; TIA, transient ischaemic attack; VKA, vitamin K antagonist.


	INTEGRATED CARE(N = 522)	USUAL CARE (N = 425)

Age (years), median (IQR)	76.0 (71.0–80.0)	77.0 (72.0–82.0)

Female sex	236 (45.2)	211 (49.6)

Hypertension	308 (59.0)	230 (54.1)

Diabetes mellitus	130 (24.9)	110 (25.9)

Prior stroke/TIA	81 (15.5)	49 (11.5)

Coronary artery disease	93 (17.8)	73 (17.2)

Prior myocardial infarction	36 (6.9)	28 (6.6)

Heart failure	72 (13.8)	66 (15.5)

Peripheral vascular disease	35 (6.7)	29 (6.8)

Prior venous thromboembolism	25 (4.8)	10 (2.4)

Chronic renal impairment	59 (11.3)	61 (14.4)

Chronic obstructive pulmonary disease	71 (13.6)	62 (14.6)

History of cancer	94 (18.0)	82 (19.3)

Pacemaker	34 (6.5)	36 (8.5)

Frailty index, median (IQR)	0.14 (0.11–0.22)	0.14 (0.11–0.19)

Polypharmacy (≥5 chronic drugs)	134 (25.7)	86 (20.2)

Anticoagulant use		

*VKA*	386 (73.9)	340 (80.0)

*NOAC*	83 (15.9)	57 (13.4)

Antiplatelet therapy	48 (9.2)	22 (5.2)

Beta-blockers	373 (71.5)	312 (73.4)

Calcium channel antagonists	149 (28.5)	111 (26.1)

Digoxin	96 (18.4)	79 (18.6)

Class I and III antiarrhythmic drugs	32 (6.1)	31 (7.3)

Diuretics	194 (37.2)	186 (43.8)

RAAS-inhibitors	278 (53.3)	248 (58.4)


### Missing data

In the intervention group, 445 out of 522 patients (85%) filled out the questionnaire at baseline, 345 out of 510 (68%) completed the questionnaire after 1 year and 305 out of 488 (63%) completed the final questionnaire after 2 years. In the usual care group, 369 out of the 425 patients (87%) who provided informed consent for the questionnaires filled out the questionnaire at baseline, 301 out of 411 (73%) completed the questionnaire after 1 year and 253 out of 397 (64%) after 2 years. Of the questionnaires that were filled out, 97% contained a complete EQ5D sub-questionnaire and in 96% the questions on home care consumption were answered completely, for example. Data on consultations and procedures in primary care were available for 19 out of 26 practices.

### Costs of health care utilisation

The costs of unadjusted and imputed costs are shown in Table 2. During the 2-year follow-up, the total costs per patient in the intervention group were € 18,845.16 compared to € 20,262.72 in the usual care group. Except for telephone consultations and small surgery/injections/ambulant compression therapy, costs from consultations in primary care were higher in the intervention group compared to usual care. For almost all other cost categories, reductions in costs in the intervention group were observed, except for the number of days admitted to the hospital, day treatment procedures, use of day care institutions and ambulance rides. The largest difference was observed for the other direct costs (unadjusted difference up to –€1,623.40 per patient over 2 years), predominantly driven by more use of assisted living facilities and home care resource use in the usual care group. The number of INR measurements did not differ between the intervention and usual care group.

**Table 2:** **Imputed, unadjusted costs for the intervention versus usual care.** Mean number of procedures and costs (in euros) per patient throughout the 2-year follow-up period. ‡ For hospital admissions, the mean length of stay in days (summed for all admissions per patient) is shown. Except for the number of INR measurements in the usual care group (for which an assumption was made, see text), all number of procedures were observed or, if indicated with *, derived through questionnaires. ^¥^ The bootstrap p-value was calculated as the proportion of bootstraps in which the mean value in the intervention arm was higher than the mean value in the control arm. ECG = Electrocardiography; ACT = Ambulant compression therapy for crural ulcers; ECV = electrocardioversion.


	TYPE OF PROCEDURE	INTEGRATED CARE (N = 522)			USUAL CARE (N = 425)			DIFFERENCE IN MEAN COSTS	BOOTSTRAPPED P-VALUE^¥^
	
		MEAN NUMBER OF PROCEDURES	MEAN COSTS	SD	MEAN NUMBER OF PROCEDURES	MEAN COSTS	SD		

PRIMARY CARE COSTS	GP consults								

	Consults	12.73	€ 120.05	*€ 5.75*	9.11	€ 86.04	*€ 3.49*	€ 34.01	*1.00*

	Double consult	4.11	€ 271.25	*€ 12.41*	2.41	€ 158.85	*€ 11.21*	€ 112.40	*1.00*

	Home visits	7.17	€ 358.52	*€ 35.92*	3.21	€ 160.37	*€ 14.71*	€ 198.15	*1.00*

	Telephone consults	6.09	€ 103.54	*€ 5.93*	6.45	€ 109.59	*€ 7.66*	–€ 6.04	*0.27*

	Practice nurse consults (chronic conditions)	4.78	€ 90.53	*€ 7.95*	0.74	€ 64.22	*€ 7.82*	€ 26.31	*0.99*

	Practice nurse consults (mental health)	0.09	€ 4.78	*NA*	0.01	€ 0.74	*€ 0.50*	€ 4.04	*0.93*

	ECG	0.19	€ 8.56	*€ 1.41*	0.10	€ 4.04	*€ 1.71*	€ 4.53	*0.97*

	Small surgery, injections, ACT	0.52	€ 31.73	*€ 4.48*	0.70	€ 46.17	*€ 10.39*	–€ 14.43	*0.10*

	Other	49.16	€ 50.21	*€ 2.57*	44.29	€ 41.88	*€ 1.73*	€ 8.33	*1.00*

	*Subtotal primary care costs*		€ 1,039.18	*€ 41.60*		€ 671.88	*€ 30.46*	€ 367.30	*1.00*

CARDIOLOGY OUTPATIENT CLINIC VISITS			€ 99.05	*€ 5.34*		€ 119.02	*€ 5.96*	–€ 19.97	*0.02*

ANTICOAGULANT TREATMENT COSTS			€ 2,174.48	*€ 34.38*		€ 2,279.26	*€ 33.34*	–€ 104.78	*0.01*

ADMISSIONS AND ECV	Hospital admissions‡	4.16	€ 1,980.60	*€ 127.30*	3.79	€ 1,804.32	*€ 176.85*	€ 176.28	*0.32*

	Temporary nursing home admissions	3.88	€ 652.05	*€ 260.55*	4.48	€ 753.04	*€ 198.18*	–€ 100.99	*0.70*

	ECV	0.09	€ 17.61	*€ 3.32*	0.11	€ 21.62	*€ 5.56*	–€ 4.02	*0.27*

	*Subtotal admissions and ECV*		€ 2,650.25	*€ 334.14*		€ 2,578.98	*€ 313.32*	€ 71.27	*0.55*

	Other outpatient visits*	6.97	€ 445.13	*€ 54.74*	7.09	€ 481.17	*€ 54.37*	–€ 36.03	*0.43*

	Day treatment*	2.06	€ 569.13	*€ 92.09*	2.10	€ 580.44	*€ 109.35*	–€ 11.31	*0.47*

	Paramedic consults*	17.96	€ 570.21	*€ 77.98*	24.83	€ 798.55	*€ 95.66*	–€ 228.34	*0.02*

	Home care*	140.59	€ 6,047.90	*€ 1,108.93*	144.81	€ 6,391.06	*€ 1,263.16*	–€ 343.17	*0.42*

	Day care institution*	4.42	€ 1,016.94	*€ 288.48*	3.67	€ 700.52	*€ 302.35*	€ 316.42	*0.80*

	Emergency department visit*	1.02	€ 264.16	*€ 52.03*	1.02	€ 263.27	*€ 47.63*	€ 0.89	*0.50*

	Ambulance ride*	0.73	€ 375.30	*€ 85.29*	0.66	€ 341.48	*€ 77.10*	€ 33.82	*0.61*

	Assisted living facility*	15.98	€ 2,684.22	*€ 913.75*	24.05	€ 4,039.91	*€ 1,263.32*	–€ 1,355.69	*0.16*

	*Subtotal other direct costs*		€ 11,972.99	*€ 1,464.16*		€ 13,596.39	*€ 1,853.53*	–€ 1,623.40	*0.23*

INDIRECT COSTS	Informal care*	235.46	€ 3,083.70	*€ 286.63*	220.26	€ 3,296.45	*€ 321.76*	–€ 212.75	*0.30*

	**Total**		**€ 18,845.16**	*€ 1,677.69*		**€ 20,262.72**	*€ 2,144.58*	**–€ 1,417.56**	*0.21*


### QALYs

Mean EQ5D-5L utility scores at baseline and after 12 and 24 months of follow-up are shown in Table 3, together with the QALY contributions. Utility scores were slightly higher in the intervention group compared to the usual care group and, in both groups, decreased during follow-up. The adjusted mean QALY contribution over 2 years was similar in the integrated care group compared to the usual care group (1.428 versus 1.429).

**Table 3:** Imputed EQ5D-5L at different time points and the QALY contribution over 2 years for the intervention versus control group.


		INTEGRATED CARE (N = 522)	SD	USUAL CARE (N=425)	SD	DIFFERENCE	BOOTSTRAP P-VALUE

IMPUTED TIMEPOINTS	T0	0.766	*0.009*	0.756	*0.019*	0.011	*0.22*

	T1	0.718	*0.013*	0.706	*0.018*	0.012	*0.25*

	T2	0.676	*0.014*	0.662	*0.010*	0.014	*0.23*

QALY CONTRIBUTION OVER TWO YEARS	QALY contribution unadjusted, censored patients included	1.439	*0.023*	1.416	*0.022*	0.022	*0.23*

	QALY contribution adjusted, censored patients included	1.428	*0.020*	1.429	*0.019*	0.000	*0.37*


### Incremental costs and effects

In Table 4, the results of the cost-utility analysis (with the mean differences between the intervention and usual care for the different adjusted and imputed cost categories and QALYs) are presented, together with their 95% percentile intervals. The number of consultations provided in the intervention group and, hence, costs in primary care were higher (up to €363 per intervention patient). In all other cost categories, the mean differences indicated lower costs in the intervention group. This resulted in a mean total cost reduction between –€865 and –€1,343 euro.

**Table 4:** **Results of the cost-utility analyses of the integrated care intervention compared to usual care.** Adjusted = for baseline differences in age, sex, Frailty Index and clustering. QALYs were also adjusted for differences in baseline EQ5D-5L utility score. Δ is the mean difference between intervention – usual care patients of 100 bootstrapped samples. The colours correspond to the different colours in Figure 2, indicating whether patients who were censored due to permanent nursing home admission, and their follow-up time while admitted to the nursing home, were included or not. ECV=electrocardioversion.


	Δ COSTS IN PRIMARY CARE	Δ CONSULTS CARDIOLOGIST	Δ ANTICOAGULANT COSTS	Δ ADMISSIONS AND ECV	Δ OTHER DIRECT COSTS	Δ INDIRECT COSTS	Δ PERMANENT NURSING HOME ADMISSION	Δ TOTAL COSTS, INCLUDING ASSUMED COSTS PERMANENT NURSING HOME ADMISSION	Δ TOTAL COSTS, WITHOUT ASSUMED COSTS FOR PERMANENT NURSING HOME ADMISSION	Δ EFFECTS (QALYS) INCLUDING PERMANENT NURSING HOME ADMISSION	Δ EFFECTS (QALYS) EXCLUDING PERMANENT NURSING HOME ADMISSION

**Base case**											

Imputed and adjusted											
522 vs 425 patients	€ 363	–€ 20	–€ 105	–€ 34	–€ 941	–€ 128	–€ 478	–€ 1,343	–€ 865	0.002	0.000
Bootstrapped 95% PI*	254:435	–32:–1	–209:–24	–737:1037	–5532:2726	–915:654	–1226:138	–6534:3109	–5730:3641	–0.035:0.046	–0.040:0.042

**Sensitivity analyses**											

522 vs 704 patients, imputed & adjusted	€ 375	–€ 17	–€ 54	–€ 337	–€ 1,648	–€ 1,013	–€ 1,175	–€ 3,868	–€ 2,693	0.049	0.060
Bootstrapped 95% PI*	251:431	–27:1	–168:8	–932:718	–7618:1523	–1957:–243	–1904:383	–9973:–50	–8799:934	0.028:0.117	0.018:0.104
Imputed, unadjusted	€ 337	–€ 17	–€ 115	€ 71	–€ 1,548	–€ 213	–€ 521	–€ 2,006	–€ 1,485	0.022	0.010
Bootstrapped 95% PI*	243:427	–33:–1	–210:–19	–816:994	–5973:2670	–1017:594	–1303:104	–7273:2878	–6606:3346	–0.039:0.084	–0.044:0.074
Healthcare perspective**	€ 151	–€ 20	–€ 105	–€ 34	–€ 941	na	–€ 478	–€ 1,427	–€ 949	0.000	0.000
Bootstrapped 95% PI*	112:193	–32:–2	–209:–24	–737:1037	–5532:2726		–1226:138	–6594:2930	–5904:3452	–0.035:0.046	–0.040:0.042


* Bootstrapped 95% percentile intervals (contains 95% of the repeats). ** In the healthcare/third party payer perspective, informal care is not included and different unit costs for primary care consultations are used, as is specified by the Dutch Health Authority (see additional file, Table A2) [18]. Unit costs in the healthcare perspective are lower per consultation compared to unit costs in the societal perspective, as the residual costs are reimbursed separately through a fixed price per registered patient.

As stated previously, the difference in effects (QALYs) between the intervention and control group was very small, between 0 and 0.002 (i.e. between 0 and 0.73 extra days alive with perfect quality of life per patient over the 2 years).

### Additional analyses permanent nursing home admission

In the control group, 8 out of 425 patients (1.9%) permanently moved to a nursing home, compared to 5 out of 522 patients (1.0%) in the intervention group. When including the remaining follow-up time assuming patients stayed alive at very low quality of life, the difference in total costs between the intervention and control group was higher (–€ 1,343 versus –€ 865) and the QALY gain similar (0.002 instead of 0.000).

### Cost-effectiveness plane

**Figure 2:**
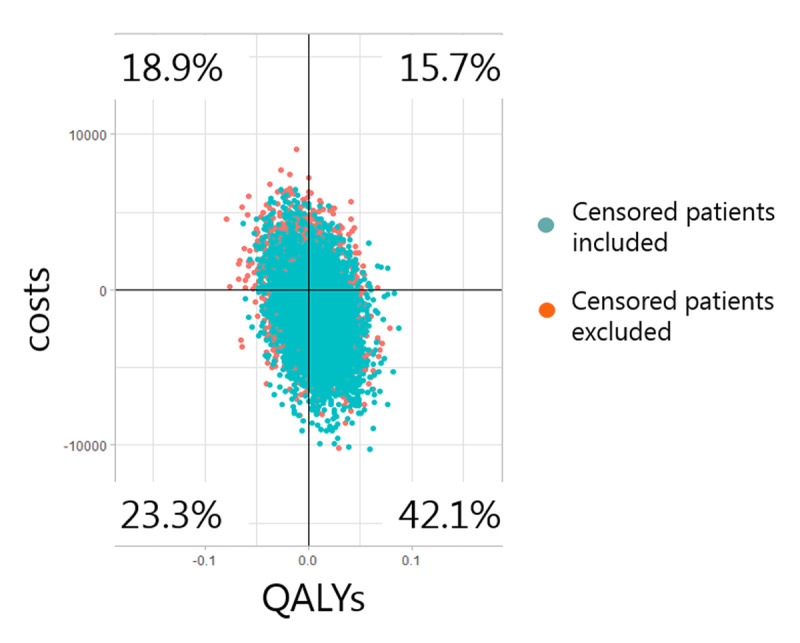
**Cost-effectiveness plane.** This figure shows the incremental costs (on the Y-axis) and incremental QALYs (on the X-axis) of integrated care compared to usual care of all the bootstrapped samples and, as is shown with the different colours, for the analyses with and without patients who were censored due to permanent nursing home admission. Negative costs (on the Y-axis) indicate cost-savings of integrated care compared to usual care, while positive costs (on the Y-axis) indicate additional spending. Negative QALYs (on the X-axis) indicate loss of QALYs due to integrated care compared to usual care, while positive QALYs (on the X-axis) indicate QALYs gained. The southeast quadrant therefore indicates the intervention to be dominant, i.e. more effective and less costly.

The cost-effectiveness plane is shown in Figure 2. Because the difference in QALYs between intervention and usual care was close to zero, the results ended up quite centred on the X-axis. 65.4% of the bootstrapped samples are in the southern part of the figure, indicating a cost reduction. Of the quadrants, the southeast quadrant (lower costs and QALYs gained) had the highest proportion, indicating a 42.1% probability of the intervention being ‘dominant’. The results of the additional analyses regarding the in- or exclusion of censored patients, and their assumed costs and effects in the remaining follow-up time after permanent nursing home admission, show a large overlap in the incremental costs and effects of the bootstrapped samples (depicted in blue and orange). The cost-effectiveness probability was defined as being more effective and cost-saving or more effective and an ICER beneath the willingness-to-pay threshold of €20.000 to €80.000. The cost-effectiveness probability was 51% to 54% in the group with censored patients included and 36% to 40% with censored patients excluded.

### Sensitivity analyses

Results of the sensitivity analyses are shown in Table 4. When including all 704 eligible usual care patients (and imputing the missing data from the questionnaires for 279 of these patients) the total cost-reduction was significantly larger: between –€ 3,868 and –€ 2,693. The QALY gain was slightly larger: between 0.05 and 0.06 (i.e. between 18 and 22 extra days alive with perfect quality of life per patient over the 2 years). The cost-effectiveness plane of this sensitivity analysis showed an 89.3% probability of the intervention being more effective and less costly [see Additional file, Figure A1] and a probability of being cost-effective between 95% and 99% when taking into account a willingness-to-pay threshold of €20,000 to €80,000.

Without adjustment for age, sex, FI and clustering, differences in QALYs and total costs were larger compared to the main (adjusted) analysis. The health care perspective resulted in smaller differences in costs from primary care consultations (as expected, as lower unit costs for primary care procedures were used).

## Discussion

We have evaluated the cost-effectiveness of the ALL-IN trial, a cluster randomised trial investigating whether integrated care for patients with AF can be safely, and cost-effectively, organised in primary care. The main analysis of this cost-utility analysis shows no apparent effect on QALYs, while mean costs indicate a cost reduction for integrated care for elderly patients with AF in primary care (€865 or €1,343 per patient per 2 years, depending on whether or not permanent nursing home admissions were included). Still, uncertainty around the costs exists, resulting in a cost-effectiveness probability between 36% and 54%. In the main analysis we excluded control group patients who did not provide informed consent for questionnaires. When we imputed this information for these usual care patients in a sensitivity analysis, the cost-effectiveness probability increased to 95%-99%.

### Interpretation of results

While the integrated care intervention, as expected, led to increased costs from consultations in primary care, this appeared to be outweighed by lower costs from other resources, especially other direct costs, indirect costs and costs from permanent nursing home admissions. However, the 95% percentile intervals around these estimates were wide due to the uncertainty on other direct costs and indirect costs. This missing information occurred ‘by design’ as in this cluster randomized trial control group participants were left blinded on the true purpose of this trial, in order to prevent contamination of the intervention to control group patients. Consequently, control group participants were less willing to fill in questionnaires on healthcare utilization and EQ5D, especially the more frail patients. This is reflected in the baseline characteristics [see Additional file, Table A3]: the usual care patients who did not provide informed consent for the questionnaires indeed appeared to be older and less healthy than those who did. We chose to only include the 425 usual care patients who were sent the questionnaires in our main analysis, as multiple imputation of the large number of missing data might have raised validity concerns. Still, in the intervention arm even more patients (404, see flow-chart) were excluded due to missing informed consent, although this consent was given with a different purpose (i.e. participating in the intervention). We could therefore argue that the “true” result, if we would have had few missing data, would at least lie between the results of the main and sensitivity analysis, where the main analysis can be regarded as more conservative since costs from hospitalisations and other (observed) cost categories from the 279 less healthy, more frail usual care patients were not taken into account.

Imputing the missing information increased the uncertainty for all our analyses. Still, because patients following the intervention were frequently monitored and treated for comorbidities including heart failure, these patients could have experienced less functional decline than patients in the control group. Although the mean costs indeed indicated a reduction in costs for e.g. home care and nursing home admission in intervention participants, this was not reflected in the results on quality of life in our main analysis, which doesn’t show a clear difference between the groups. The overall cost-effectiveness probability was attenuated by this lack of effect on QALYs, ending up between 36% and 54%. Still however, a quality of life difference was shown in the sensitivity analyses including (with imputation) *all* usual care patients, resulting in a cost-effectiveness probability between 95% and 99%. Combined with the observed reduction in use of home care and assisted living facilities and the reduction in all-cause mortality, this supports the theory of less functional decline due to integrated AF management. In addition, Bleijenberg and colleagues also reported a, rather small, effect on functional decline and reduced costs due to fewer days of nursing home admissions and fewer hours of informal care among frail elderly receiving nurse-led care, compared to usual care [19,20].

Remarkably, in our data, cardiology outpatient clinic consultations contributed relatively little to the difference in total costs. This can be explained by the observation that also in usual care a large proportion (52%) of patients had already been discharged from routine outpatient cardiology follow-up, decreasing potential substitution of care.

### Strengths and limitations

An important strength of this cost-effectiveness study is that we included data from a broad range of resources, ranging from informal care to hospital care. Furthermore, most of the resources consisted of actually observed data from our trial. Nevertheless, the following limitations need to be noted. First, as explained in detail above, the main limitation is that data on quality of life and self-reported health care consumption were missing for about 40% of the 704 usual care patients who did not provide informed consent for the questionnaires, as a consequence of the trial design. A second limitation is that we did not have the exact number of INR measurements per patient in the usual care group because of the informed consent procedure. Third, for the same reason, we had to censor patients after permanent nursing home admission. Because the admission rate might have been affected by the intervention, we decided to make extreme assumptions on the duration of stay to display the potential influence of these censored patients on the outcome.

Lastly, in the intervention group, the increase in GP consultations was larger than the increase in practice nurse consultations, likely caused by the difficulty to distinguish between practice nurse and GP consultations in our data. For reimbursement reasons, a practice nurse consultation is sometimes registered as a GP consultation [21].

### Comparison to existing literature

The results of this cost-effectiveness study are in line with the results from Hendriks and colleagues, who investigated the cost-effectiveness of integrated nurse-led care at a specialised AF clinic of a tertiary care hospital in the Netherlands [9]. Although performed from a hospital perspective, disregarding costs from primary care and informal care, they observed a cost reduction of €1109 per patient per year and a mean QALY gain of 0.009 (no 95% PIs reported). We observed a QALY gain between 0.000 and 0.002 and a cost reduction between €433 and €672 per patient per year (depending on whether costs of permanent nursing home admission were included). In other countries, examples of integrated care initiatives for AF patients have also shown promising results. In Australia, a randomised study comparing an AF-specific, nurse-led, home-based intervention to usual post-discharge care, revealed a small increase in QALYs (0.02 per person) and a reduction in total healthcare costs (4,375 Australian dollars per person over 1.75 years) [22]. Other non-randomized studies aimed at risk management performed in patients with AF in Australia, Canada and Italy have all shown small QALY gains and substantial cost reductions [23,24,25]. Although these studies were quite heterogeneous, it appears that the common ingredient of frequent follow-up with treatment of comorbidities forms the basis of managing the increasing health care burden associated with AF. Our study is currently the only randomized study organised from primary care with a generalist instead of AF-specific approach as well as a societal perspective. Studies evaluating other nurse-led care programs for non-AF patients in primary care, regarding for example heart failure, frail elderly, or cardiovascular risk management, have also observed cost reductions and QALY maintenance or gains [20,26,27].

### Clinical implications and future considerations

This cost-effectiveness study, together with the observed reduction in mortality as presented previously [11], provides valuable information for policy makers and healthcare insurers to guide further implementation of integrated care for AF patients. Currently, substitution of care from secondary to primary care is a popular strategy in managing the increasing disease burden of an ageing society. It is important to emphasize that the ALL-IN trial was aimed at *integration* rather than *substitution* of care, as the intervention had a multidisciplinary nature with (if appropriate) check-ups in secondary care in addition to check-ups in primary care. In the usual care group, one third of all patients did not receive any proactive cardiovascular follow-up. Therefore, a considerable number of patients following our intervention received *extra* care, which likely explains the beneficial results on mortality. Moreover, shared care better meets the complex needs of AF patients, especially in those who suffer from severe (cardiac) comorbidity [27,28]. It is increasingly recognized that AF is part of a complex interplay of multiple cardiovascular and non-cardiovascular comorbidities [29,30,31]. It is therefore important to integrate treatment of these comorbidities in the treatment of AF, as is also stated in the 2020 ESC Guidelines for the diagnosis and management of AF [32]. As the broad, holistic approach is ‘in the DNA’ of primary care, and because costs in primary care are substantially lower compared to secondary care, primary care still forms an attractive setting for further implementation of integrated care for AF patients.

When considering further implementation and future research, joint (or video)consultations between cardiologists and general practitioners in certain more complex patients might be a promising development to enable shared care while reducing referrals to secondary care [33]. In this way, a more evident cost-reduction might be realised. The use of e-health technology has also shown promising results in AF patients, while increasing patient involvement [34].

For future policy making, the GP perspective is also important, as the increase in consultations could make implementation costly for primary care practices. In the Netherlands, reimbursement per consultation is substantially lower than the estimated unit costs (approximately 1/3). The residual reimbursement is paid to the GP as a fixed amount per registered patient, which becomes relatively low when the number of consultations increases. Although reimbursement structures differ per country, we expect the intervention and its effects to be transferrable to many other countries with a primary care setting. In fact, primary care practices are often located much closer to home than hospitals, especially in larger countries. Offering integrated AF care closer to the patient’s home could therefore increase accessibility for patients. Future studies evaluating integrated AF care in different countries, including also travel costs and joint (video)consultations, are therefore desired.

## Conclusion

The higher costs from extra primary care consultations were likely outweighed by cost reductions for other resources, while no apparent effect on QALYs was seen. However, the main analysis showed a low cost-effectiveness probability of 36% to 54%. It is likely that the results were influenced by the limitation that a large part of control patients had missing data on quality of life, since imputing these patients in the sensitivity analysis resulted in a high probability for cost-effectiveness. Therefore, this study does not give sufficient clarity yet on the cost-effectiveness of integrated care compared to usual care for AF patients.

## Additional File

The additional file for this article can be found as follows:

10.5334/ijic.5661.s1 Additional file. Detrended Oscillation and Clock Parameters.
