# Supplementary material for: Managing the Increasing Burden of Atrial Fibrillation through Integrated Care in Primary Care: A Cost-Effectiveness Analysis
Source: Int J Integr Care. 2023 May 3;23(2):9. doi: 10.5334/ijic.5661 (PMC10162350; doi:10.5334/ijic.5661)
Supplement: Additional file. — Detrended Oscillation and Clock Parameters. [file ijic-23-2-5661-s1.pdf]

Additional file

Table A1. Detailed description of the intervention in comparison to usual care

	**Integrated care intervention in primary care**	**Usual care**
**Description**	**1. Quarterly follow-up visits:**<br>Proactive, structured face-to-face check-ups for AF treatment and treatment and prevention of cardiovascular and non-cardiovascular comorbidities, according to a check-list (see appendix) based on Dutch primary care guidelines on AF and ESC guidelines. Visits included special attention to detection of signs of heart failure, adequate rate control, evaluation of the need for laboratory testing or ECG (available within the primary care practices), lifestyle improvement and patient education/empowerment.<br>**2. Tailored anticoagulation monitoring:**<br>*In VKA patients:* regular INR measurements in the primary care practice or if necessary at home. The INR value was measured from a capillary blood sample with the Coaguchek® and communicated together with information on important interacting factors (e.g. fever, pain, medication or dietary changes) to the anticoagulation clinic through an online portal. The anticoagulation clinic acted like a 'back-office' and created the dosage calendar that was sent back to the primary care practice or to the patient. Some patients measured the INR themselves at home.<br>*In NOAC patients:* adherence, patient education, and kidney function monitoring were part of the checklist of the quarterly follow-up visits.<br>**3. Close collaboration with specialists:**<br>Cardiologists and anticoagulation clinics were easily accessible for consultation. At the start of follow-up, cardiologists were asked to evaluate if the patient could be discharged from outpatient cardiology care or not. If not, cardiology care was complementary to the integrated care intervention in primary care.	Variable and delivered by different health care professionals (cardiologists, specialised AF nurses, anticoagulation clinics, general practitioners, practice nurses), without one designated coordinator (see text). Some patients did not receive any care during follow-up. In VKA patients, INR measurements were performed by anticoagulation clinics by venepuncture. Some patients measured the INR themselves at home.
**When and how much**	Prescheduled quarterly follow-up visits to the primary care practice (3 times a year with practice nurse, once yearly with GP). In addition, VKA-patients received INR measurements, about 20 times a year.	Usually ad-hoc visits to the GP when symptoms have developed. In some patients pre-planned visits to the cardiology outpatient service once or twice a year. VKA-patients received INR measurements, on

		average about 20 times a year.
**Who provided AF care**	The practice nurse, supervised by the GP, was the predominant deliverer of integrated AF care. To ensure continuity during holidays for example, practice assistants were also trained to perform INR measurements.	Variable (cardiologists, specialised AF nurses, GPs, practice nurses, anticoagulation clinic personnel, or no one).
**Training**	At the start of the trial, participating practice nurses, assistants and general practitioners received 4 hours of training, given by the corresponding author, cardiologists, and the anticoagulation clinic. Education included treatment of AF and its related comorbidities, anticoagulation monitoring (including information on when to perform extra INR measurements) and referral criteria. Evaluation meetings were organised 3 times during the 2 year follow-up, to share knowledge, practical issues and interesting or complicated cases.	No specific training about AF. In the Netherlands, practice nurses have a post-bachelor degree in chronic disease management of diabetes, COPD and cardiovascular risk management, yet without specific attention to AF.
**Initiator/ coordinator of care**	The practice nurse.	Usually the patient (and/or cardiologist when treated in out-patient cardiology clinic, and/or anticoagulation clinic).
**Scope of care**	Holistic.	AF-focused.
**Location of care delivery**	Primary care practice (or when necessary at the patient's home).	Variable (out-patient cardiology clinic, anticoagulation clinic, primary care practice).
**Responsibility**	Shared responsibility, but primarily the GP (although anticoagulation clinics remained responsible for the VKA dosing calendar).	Often unclear or variable (cardiologist, GP, anticoagulation clinic).
**Patient education**	Information on AF, complications, importance of adherence to anticoagulants and when to contact the primary care practices were given to all eligible patients by the GP during the recruitment visit. In large practices we organised an information evening together with the practice. All eligible patients received a 15 page booklet with information on AF and the trial. Importantly, patients actively participating in the intervention received education (especially on the importance of VKA/NOAC adherence) repeatedly during follow-up visits.	No specific patient education was provided.
**Communication**	Easy access communication was encouraged between patients, GPs and practice nurses within the practices; with the anticoagulation clinic through the online portal; and with cardiologists and specialised AF nurses in secondary care through telephone or pre-existing digital and secured communication systems.	No specific agreements were made regarding communication.

**Clinical example of short lines of communication**	A 90-year old AF patient is visited at home by her GP because of pneumonia. The GP prescribes an antibiotic and asks the practice nurse to perform an extra INR measurement that same day, which appeared to be too high. After consulting the anticoagulation clinic, vitamin K was prescribed and a short-term follow-up INR measurement was planned.	Some VKA users know that they should contact the anticoagulation clinic when they feel ill, but many patients only contact their GP. Out-of-range INR levels are then only detected during regular INR measurements, or worse, when a bleeding occurs.

GP, general practitioner; ESC, European Society of Cardiology; ECG, electrocardiography; VKA, vitamin K antagonist; NOAC, non-vitamin K antagonist oral anticoagulant; INR, International Normalised Ratio; COPD, chronic obstructive pulmonary disease.

Table A2. Differences in unit costs of consultations between societal and healthcare perspective

Type of procedure	Societal perspective (base case analysis)	Healthcare perspective (sensitivity analysis)
Consultation ≤ 5 minutes	€ 33.00	€ 4.98
Consultation 5 - 20 minutes	€ 33.00	€ 9.97
Consultation ≥ 20 minutes	€ 66.00	€ 19.94
Home visit	€ 50.00	€ 14.95
Consultation by phone	€ 17.00	€ 0.00
ECG costs	€ 45.18	€ 45.18
Check-up by a primary care practice nurse	€ 33.00	€ 1.00

Unit costs of consultations in primary care according to the Dutch Manual for costing research in health care (Hakkaart-van Roijen et al., 2015) as used in the base case analysis with a societal perspective and as used in the sensitivity analysis with a healthcare/third party payer perspective, as specified by the Dutch Health Authority. (Nederlandse Zorgautoriteit, 2020) Unit costs in the healthcare perspective are lower per consultation compared to unit costs in the societal perspective, as the residual costs are reimbursed separately through a fixed price per registered patient.

Table A3. Baseline characteristics of included patients

	Integrated care (n = 522)	Usual care	
		All eligible patients (n = 704)	*With informed consent for questionnaires (n=425)*
Age (years), median (IQR)	76.0 (71.0-80.0)	78.0 (72.0-83.0)	*77.0 (72.0-82.0)*
Female sex	236 (45.2)	369 (52.4)	*211 (49.6)*
Hypertension	308 (59.0)	386 (54.8)	*230 (54.1)*
Diabetes mellitus	130 (24.9)	182 (25.9)	*110 (25.9)*
Prior stroke/TIA	81 (15.5)	95 (13.5)	*49 (11.5)*
Coronary artery disease	93 (17.8)	119 (16.9)	*73 (17.2)*
Prior myocardial infarction	36 (6.9)	49 (7.0)	*28 (6.6)*
Heart failure	72 (13.8)	132 (18.8)	*66 (15.5)*
Peripheral vascular disease	35 (6.7)	47 (6.7)	*29 (6.8)*
Prior venous thromboembolism	25 (4.8)	30 (4.3)	*10 (2.4)*
Chronic renal impairment	59 (11.3)	108 (15.3)	*61 (14.4)*
Chronic obstructive pulmonary disease	71 (13.6)	97 (13.8)	*62 (14.6)*
History of cancer	94 (18.0)	128 (18.2)	*82 (19.3)*
Pacemaker	34 (6.5)	62 (8.8)	*36 (8.5)*
Frailty index, median (IQR)	0.14 (0.11-0.22)	0.17 (0.11-0.19)	*0.14 (0.11-0.19)*
Polypharmacy (≥5 chronic drugs)	134 (25.7)	139 (19.7)	*86 (20.2)*
Anticoagulant use			
VKA	386 (73.9)	564 (80.1)	*340 (80.0)*
NOAC	83 (15.9)	79 (11.2)	*57 (13.4)*
Antiplatelet therapy	48 (9.2)	50 (7.1)	*22 (5.2)*
Beta-blockers	373 (71.5)	516 (73.3)	*312 (73.4)*
Calcium channel antagonists	149 (28.5)	181 (25.7)	*111 (26.1)*
Digoxin	96 (18.4)	135 (19.2)	*79 (18.6)*
Class I and III antiarrhythmic drugs	32 (6.1)	52 (7.4)	*31 (7.3)*
Diuretics	194 (37.2)	336 (47.7)	*186 (43.8)*
RAAS-inhibitors	278 (53.3)	393 (55.8)	*248 (58.4)*

Numbers are counts (%) unless stated otherwise. The frailty index consists of the presence or absence of 36 health deficit items (scale 0–1, higher value indicating more frailty). EQ5D-5L, EuroQol 5D questionnaire; IQR, interquartile range; NOAC, non-vitamin K antagonist oral anticoagulant; RAAS, renin–angiotensin–aldosterone system; TIA, transient ischaemic attack; VKA, vitamin K antagonist.

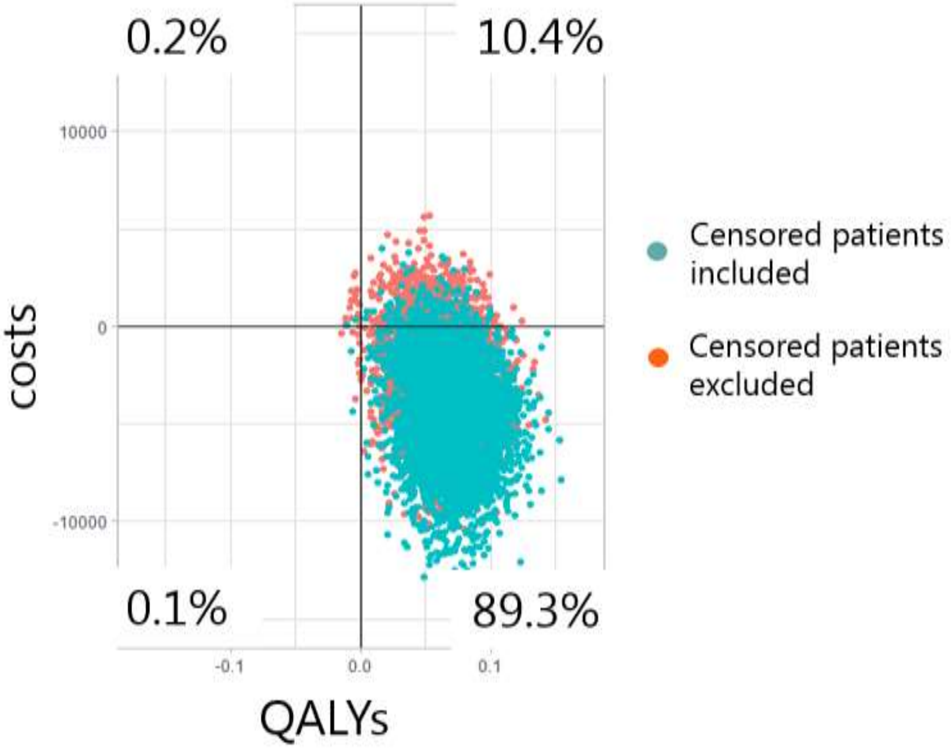

Figure A1 *Cost-effectiveness plane sensitivity analysis all eligible usual care patients*
This figure shows the incremental costs (on the Y-axis) and incremental QALYs (on the X-axis) of the 522 integrated care patients compared to the 704 eligible usual care patients of all the bootstrapped samples and, as is shown with the different colours, for the analyses with and without patients who were censored due to permanent nursing home admission. Negative costs (on the Y-axis) indicate cost-savings of integrated care compared to usual care, while positive costs (on the Y-axis) indicate additional spending. Negative QALYs (on the X-axis) indicate loss of QALYs due to integrated care compared to usual care, while positive QALYs (on the X-axis) indicate QALYs gained. The southeast quadrant therefore indicates the intervention to be dominant, i.e. more effective and less costly.
